# “My Body, My Rhythm, My Voice”: a community dance pilot intervention engaging breast cancer survivors in physical activity in a middle-income country

**DOI:** 10.1186/s40814-023-01253-x

**Published:** 2023-02-28

**Authors:** María Alejandra Rubio, Carlos M. Mejía-Arbeláez, Maria A. Wilches-Mogollon, Sergio Moreno, Carolyn Finck, Lisa G. Rosas, Sol A. Romero, Paula Guevara, Santiago Cabas, Oscar Rubiano, Alberto Flórez-Pregonero, José G. León, Luis Fernando Alarcón, Robert Haile, Olga L. Sarmiento, Abby C. King

**Affiliations:** 1 School of Medicine, Universidad de los Andes, Carrera 1, #18ª-12, Bogotá, Colombia; 2 School of Engineering, Universidad de los Andes, Carrera 1 #18ª-12, Bogotá, Colombia; 3 Department of Psychology, Universidad de los Andes, Carrera 1 #18ª-12, Bogotá, Colombia; 4 Department of Epidemiology and Population Health, Stanford University School Medicine, Stanford, CA 94305 USA; 5 Division of Primary Care and Population Health, Department of Medicine, Stanford University School of Medicine, Stanford, CA 94305 USA; 6 Faculty of Health Sciences, Universidad Manuela Beltrán, Bogotá, Colombia; 7 Research Department, Universidad Libre, Bogotá, Colombia; 8 School of Education, Pontificia Universidad Javeriana, Bogotá, Colombia; 9 Sports Medicine Service, Hospital de San José, Bogotá, Colombia; 10 Cedars-Sinai, Los Angeles, USA; 11 Stanford Prevention Research Center, Department of Medicine, Stanford University School of Medicine, Stanford, CA 94305 USA

**Keywords:** Breast cancer survivors, Physical activity, Behavior change intervention, Community-based program, Mixed-method assessment, Middle-income setting

## Abstract

**Background:**

Interventions to promote physical activity among women breast cancer survivors (BCS) in low- to middle-income countries are limited. We assessed the acceptability and preliminary effectiveness of a theory-driven, group-based dance intervention for BCS delivered in Bogotá, Colombia.

**Methods:**

We conducted a quasi-experimental study employing a mixed-methods approach to assess the 8-week, 3 times/week group dance intervention. The effect of the intervention on participants’ physical activity levels (measured by accelerometry), motivation to engage in physical activity, and quality of life were evaluated using generalized estimating equation analysis. The qualitative method included semi-structured interviews thematically analyzed to evaluate program acceptability.

**Results:**

Sixty-four BCS were allocated to the intervention (*n* = 31) or the control groups (*n* = 33). In the intervention arm, 84% attended ≥ 60% of sessions. We found increases on average minutes of moderate-to-vigorous physical activity per day (intervention: +8.99 vs control: −3.7 min), and in ratings of motivation (intervention change score = 0.45, vs. control change score= −0.05). BCS reported improvements in perceived behavioral capabilities to be active, captured through the interviews.

**Conclusions:**

The high attendance, behavioral changes, and successful delivery indicate the potential effectiveness, feasibility, and scalability of the intervention for BCS in Colombia.

**Trial registration:**

ClinicalTrial.gov NCT05252780, registered on Dec 7th, 2021—retrospectively registered unique protocol ID: P20CA217199-9492018.

**Supplementary Information:**

The online version contains supplementary material available at 10.1186/s40814-023-01253-x.

## Key messages regarding feasibility

- What uncertainties existed regarding the feasibility?
  There is uncertainty about the implementation of a dance-based intervention to promote physical activity among breast cancer survivors (BCS) in a community setting in Colombia, as well as the assessment of the acceptability and effectiveness through a mixed-methods study.
- What are the key feasibility findings?
  The proposed intervention was successfully delivered and assessed, showing high attendance, acceptability, and behavioral changes.
- What are the implications of the feasibility findings for the design of the main study?
  The feasibility findings are promising to promote the systematic uptake and evaluation of *My Body* theory-driven intervention for breast cancer survivors in the community setting in Colombia.

## Background

Breast cancer is the most common cancer among women worldwide [1]. In Latin America, breast cancer incidence has been rising in most countries over the last decade, increasing between 2012 and 2018 from 52.1 to 56.8 new cases per 100,000 women [2, 3]. In Colombia, during the period 2011–2018, the breast cancer incidence rate increased from 33.8 to 44.1 cases per 100,000 women, and the mortality rate increased from 9.9 to 11.9 deaths per 100,000 women [4, 5]. Furthermore, the 5-year net survival for women diagnosed with breast cancer decreased in Colombia from 79.1% in 2005 to 72.2% for the women diagnosed during the 2010–2014 period [6].

A large body of evidence supports the beneficial effects of physical activity for breast cancer survivors (BCS), including improvements in survival, physical functioning, psychological complaints, and overall quality of life (QoL) [7, 8]. Worldwide evidence-based exercise guidance for cancer survivors recommends daily physical activity, as it contributes to reductions in body fat, metabolic and sex hormones, growth factors, and inflammation, as well as broader biopsychosocial outcomes (i.e., it reduces symptoms of depression/anxiety, insomnia, fatigue, and improves well-being) [9, 10]. However, many BCS are not meeting the physical activity recommendations [11]. Evidence from high-income countries shows low physical activity participation and more likelihood to engage in sedentary behavior among BCS [12, 13]. Among Colombian BCS, low physical activity levels have also been reported [14]; however, to the best of our knowledge, there are no prevalence estimates of physical activity among BCS in Colombia. We know that 57% of Colombian adult women do not meet the current guidelines [15], and the most reported barriers to regular physical activity for Colombian women include time management, caregiving roles, safety concerns, and inadequate recreational facilities [16, 17].

The challenge of adhering to an active lifestyle has led to the development and assessment of theory-driven interventions promoting physical activity tailored for BCS as a way to help individuals adopt and maintain healthy behaviors [18]. Thus far, physical activity programs tailored to the needs and preferences of BCS have been lacking in Latin America, a region characterized by a disproportionately lower prevalence of physical activity among women compared to men [19]. Behavioral interventions undertaken in the USA and other high-income countries have achieved increases in physical activity levels in BCS using diverse cognitive-behavioral techniques [20, 21]. A systematic review and meta-analysis of 27 interventions designed to promote physical activity behavior change in cancer survivors concluded that interventions with more intense supervision (i.e., in-person, frequent interactions) tended to produce larger effects on behavior change [22], and “action planning,” “graded tasks,” and “social support” were identified as promising intervention components that facilitated behavior change [22]. In addition, qualitative studies evaluating elements of program adherence success among interventions targeting BCS have underscored the group-based environment, which can facilitate social support and camaraderie of others [23] in addition to physical activity and emotional modeling that facilitates positive psychosocial well-being [24]. Group-based interventions targeting BCS have been developed mainly in high-income countries [25], suggesting its potential in regions like Latin America, where social support and other interpersonal processes have been positively related to initiating and maintaining physical activity among women [26, 27].

From an ecological perspective, behavioral interventions, in addition to targeting individuals, should also affect interpersonal, organizational, and environmental factors influencing health behavior, while addressing the question of how practitioners can best integrate theory into large-scale public health programs. Colombia is an international leader in developing innovative, publicly funded, community-based physical activity programs, some of which have been in place for more than 20 years [28], and are now being implemented in other low- and middle-income countries (LMICs), as well as higher income nations. These group-based physical activity programs have shown promise in promoting physical activity among Latin American women by incorporating dance in guided sessions [29]. However, the role of health behavior theories in the ongoing programs has not been clear or has been secondary to practical concerns. In this context, a relevant next step is to design a theory-driven intervention to promote physical activity among Colombian BCS by leveraging the existing community-based approach. A culturally tailored, group-based dance intervention to promote physical activity is one approach that appears to be feasible, acceptable, and potentially effective to increase physical activity in Latin America, as dance is a well-known motivating form of physical activity [30, 31].

The aim of the “My body, My Rhythm, My Voice” (from now on referred to as *My Body*) pilot study was to assess the acceptability and preliminary effectiveness of a theory-driven, group-based dance intervention for BCS delivered by a governmental entity in Bogotá, Colombia. Using a mixed methods approach, we aimed to (1) assess the individual-level primary outcomes for this pilot including physical activity levels, QoL, and motivation to engage in physical activity and (2) assess the acceptability of the program from a socioecological perspective, including semi-structured interviews inquiring about barriers and facilitators at the individual, interpersonal, and community levels. This study is innovative in that, to our knowledge, it is the first to pilot-test a theory-driven community-based physical activity program for BCS in Latin America. The research was conducted prior to the COVID-19 pandemic.

## Methods

### Study setting

In Colombia, a middle-income country [32], public institutions from the healthcare and sports/recreation sectors offer community-based programs aimed at physical activity promotion using a life-course approach [28, 33]. In addition, breast cancer-related stakeholders offer diverse activities as private initiatives to increase BCS’s quality of life. In the *My Body* study, we facilitated cross-sectoral partnerships between local entities to implement an 8-week evidence-based behavioral physical activity program for BCS using a community-based dance intervention. The municipal Recreovía dance-based physical activity program [29], publicly funded by Bogotá’s Sports and Recreation Institute, was in charge of *My Body* program delivery.

### Study design

The study piloted a two-armed, quasi-experimental trial comparing an intervention to a usual care control arm. This pilot study employed a convergent mixed-methods approach [34] to integrate pre- and post-quantitative and qualitative data methods. The quantitative methods included pre-test–post-test assessments. The qualitative methods included pre- and post-intervention semi-structured interviews with both intervention and control arms to understand facilitators and barriers to program acceptability. We summarized the quantitative and qualitative results in a table to gain a more thorough understanding of contextual factors that could influence the intervention outcomes. All participants signed informed consent, and the study was approved by the Universidad de los Andes ethics committee, Act Number 949 of 2018.

### The “My Body, My Rhythm, My Voice” physical activity behavioral intervention

Following a socioecological approach to promote physical activity behavior change, *My Body* was co-created through cross-sector collaboration among academic researchers and stakeholders from local and national public and private healthcare and sports/recreation institutions, healthcare service providers, and community organizations supporting cancer survivors. Members of the interdisciplinary and cross-sectoral research team (e.g., physiotherapist, epidemiologist, psychologist, respiratory therapist, physician, anthropologist) had monthly meetings (*N* = 8). Methodological details concerning the co-creation have been reported elsewhere [35]. Based on the discussions and a systematic review of the literature, we designed the 8-week, 3 times/week dancing-based physical activity intervention, mostly informed by the social cognitive and self-determination theories [36, 37]. The goal of the intervention was to gradually increase all participants’ physical activity levels to achieve significant health benefits. For this reason, the set of strategies to facilitate behavior change was deployed progressively, week by week, through the interaction of the dance-based physical activity sessions and a psychoeducational booklet designed for BCS, described as follows (online resource 1).

According to the guidelines of the American College of Sports Medicine for cancer patients [9], physical activity intensity increases were progressive and moderate. Sessions were 45 min initially, followed by 5-min increases per week, until reaching 60 min by the fifth week, plus 10-min warm-up and 10-min cool-down activities. Physical activity intensity increases were determined using maximum heart rate (HR) based on reference equations for BCS and considering the relationship between the expected exercise HR and the speed of a given song accompanying the group dance movements [38]. Based on these determinations, the playlist music was selected according to each song’s tempo (beats per minute [BPM]) and expected HR to maintain a comfortable level of exertion during the exercise [39]. Including culturally relevant dancing rhythms such as salsa and champeta, the music BPM progressively increased as shown in online resource 1*.* The instructors had been previously certified in dancing-based physical activity instruction by the Recreovía program. Participants were encouraged to invite their caregivers or another supportive person to attend the physical activity sessions with them.

The behavioral science research team members designed a psychoeducational booklet for BCS containing activities to foster behavior change (e.g., goal setting, action planning, enhancing decisional balance for being active, self-monitoring) [22, 36, 37]. The psychoeducational booklet was given to participants at the beginning of the intervention, and once per week, a researcher attended the session to follow up and guide participants’ use of the booklet and to provide motivational and behavioral instruction and support (online resource 1). The booklet was also used to train the physical activity instructors on behavior change strategies. As part of the physical activity intervention, the participants were taught by their instructor during each class how to utilize behavioral and cognitive self-regulatory skills to increase and maintain their physical activity participation (e.g., action planning, coping planning, counter conditioning, self-evaluation).

No specific instructions were given to the usual care group concerning physical activity behavior change beyond any given by their health care providers.

### Recruitment of breast cancer survivors

An a priori sample size of 30 participants per study group was based primarily on logistical and budgetary constraints for the pilot study. BCS were invited to the study through word of mouth, flyers, and social media from partner organizations. Eligible women were BCS at least 6 months post-treatment completion, more than 18 years of age, living in Bogotá, and willing to attend the program and the assessments. To enhance diversity in this first-generation study, individuals were eligible irrespective of whether they were currently meeting physical activity guidelines or not. Exclusion criteria were the presence of metastatic disease and other health conditions for which community physical activity was contraindicated. Medical clearance for participation provided by sports medicine physicians, who were members of the research team, was required. Women who did not receive physician approval were not included in the study. Given the need to respect partner organizations’ concerns about providing differential interventions to their patients through randomization, the first half of the participants with physician approval was allocated to the intervention group, while the second half was allocated to a waitlist control group. Participants in the intervention group received modest incentives in sessions 1, 12, and 24 (e.g., a water bottle, sun cap, drawstring bag), as well as a small financial remuneration for transportation to each attended community session (equalling approximately $1.25 USD per session). Participants in the control group received the physical activity intervention at the end of the 8-week study period after completing the pre-post-study assessments. Due to time and funding constraints, no outcome measurements were collected for the waitlist control group when they received their program.

### Quantitative assessment

Trained personnel evaluated participants at Universidad de los Andes’ campus facilities and at the intervention location, which was a residential community center, administered by Bogotá’s Sports and Recreation Institute. We collected accelerometry measurements, as well as self-reported questionnaires, that captured sociodemographic characteristics, medical history, and QoL (all described below). All measurements were performed at baseline (time 0) and at 8 weeks (time 1, i.e., immediately post-intervention).

#### Outcome variables

##### Physical activity levels/accelerometry

To assess participants’ physical activity levels, participants wore the Actigraph GT3X or GT3X+ Accelerometers (ActiGraph, Pensacola, FL, USA) for 7 consecutive days from awakening to bedtime using an elasticized belt around the waist at the right mid-axillary line. The accelerometer was removed when bathing or sleeping. For wear-time validation, a minimum of 4 weekdays and a weekend day with at least 10 h of wear during the waking time was required. Accelerometers were initialized to collect data at a sampling frequency of 80 Hz, downloaded in 1-s epochs, and grouped in 60-s epochs for analysis. After the data collection, we validated the time of use with an algorithm programmed in R (version 3.3.2). The data were scored using the Freedson cut-points for adults [40].

Additionally, to measure physical activity levels during dancing sessions, two participants, randomly selected during each session, wore an accelerometer with an elasticized belt around the waist at the right mid-axillary line.

##### Motivation to engage in physical activity

Self-determination theory (SDT) conceptualizes motivation as a continuum ranging from amotivation (complete lack of self-determination to perform the behavior) to a high level of intrinsic motivation (high self-determination, the behavior is driven by enjoyment) [37, 41, 42]. As part of this continuum, extrinsic motivation is differentiated as four regulatory processes that range in level of internalization and self-determination (external, introjected, identified, integrated regulation) [42]. To assess motivational regulation for physical activity, we used the validated Spanish version of the Behavioral Regulation in Exercise Questionnaire-3 (BREQ-3) [43], a 23-item inventory assessing the above SDT-relevant constructs. Responses to each item were reported on a 5-point scale ranging from 0 (not true for me) to 4 (very true for me).

##### Quality of life

To assess health-related QoL, we used the official Colombian Spanish translation of the European Organization for Research and Treatment of Cancer Quality of Life Questionnaire (EORTC QLQ-C30) [44]. It incorporates five functioning subscales (physical, role, cognitive, emotional, and social functioning), three symptom subscales (fatigue, pain, and nausea/vomiting), six single symptoms (dyspnea, appetite loss, insomnia, constipation, and diarrhea), an item for illness-related financial difficulties, and a two-item general health/global QoL subscale. Each item was reported on a 4-point scale ranging from 1 (not at all) to 4 (very much), except for the general health subscale, for which response options range from 1 (very poor) to 7 (excellent). All the scores are transformed into the range of 0–100. High scores on the functioning subscales and the global health status/QoL subscale represent higher QoL, while high scores on the symptom subscales indicate high levels of symptomatology, hence, poorer QoL. We also used a subscale of the EORTC questionnaire specific to breast cancer patients regarding physical symptoms of lymphedema; it comprises seven items with responses also ranging from 1 (not at all) to 4 (very much). We followed the questionnaire manuals to perform statistical analyses and compared results to normative values for the Colombian population [44].

#### Sociodemographic characteristics

We collected surveys inquiring about sociodemographic variables including age, education, socioeconomic level, employment status, and healthcare access.

#### Statistical analyses

Descriptive statistics (mean, standard deviation, absolute frequency, and percentage) were calculated for each outcome variable at baseline and post-intervention. To characterize the population, we compared the intervention with the control group on sociodemographics at baseline using independent *t* test or chi-square procedures depending on the nature and distribution of the variable. Generalized estimating equations (GEEs) with non-structure correlation were used to analyze changes in primary behavioral outcomes (e.g., physical activity levels) and secondary behavioral outcomes (e.g., motivation to engage in physical activity), and QoL across the intervention period occurring within subjects. GEE was used for its ability to provide a population-averaged effect from repeatedly measured data of multiple subjects in absence of normality. A coefficient interaction between the time of observation and the presence/absence of intervention and 95% confidence intervals (CI) were calculated to determine the possible effects of the intervention in this population. Stata®, version 16.0, and R Core Team, version 4.0, were used to analyze the data.

### Acceptability assessment: interviews

Women in the intervention and control groups participated in the baseline interviews, while only the intervention group participated in follow-up interviews pertaining to the acceptability of the intervention (total interviews = 37). We used a semi-structured interviewing technique to ensure in-depth insights about BCS perspectives towards physical activity, their anticipated and actual experienced barriers and facilitators to engage in the physical activity intervention, their expected and reported benefits from the physical activity intervention, and the perceived positive and negative aspects of the program.

A social scientist on the study team (MAR) led the interviews, which had an average participation of three BCS and 18-min duration each. Interviews were conducted in small groups, given the purpose of generating collective insights regarding program acceptability. All interviews were audio-recorded, transcribed verbatim, and thematically analyzed using Excel thematic matrices (Microsoft Corporation, 2018). Four researchers, who were separate from the interview facilitator, independently conducted the analysis. The total number of transcripts was divided in two, and each half was duplicated and analyzed by two researchers. Transcripts were thematically analyzed according to the socioecological model [45] using the following categories and subthemes: perceived barriers to engage in the intervention (intrapersonal, interpersonal, community level), perceived facilitators (intrapersonal, interpersonal, community level), perceived benefits (physical, mental, and social), and program recommendations. Researchers participated in weekly meetings to discuss interpretations and resolve discrepancies.

## Results

### Study recruitment and attrition

**Fig. 1:**
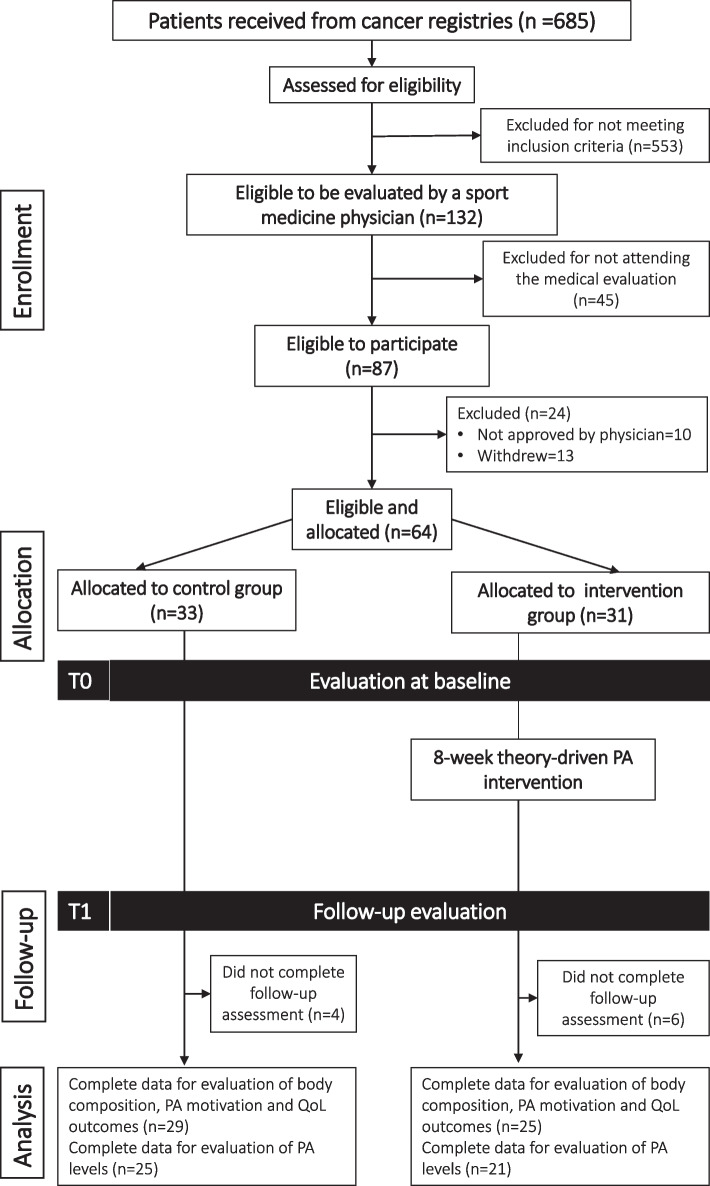
Consort flow diagram of “My body, My Rhythm, My Voice” study population

The participants were recruited from March 2019 to September 2019 (Fig. 1). The combined recruitment efforts with partner organizations yielded 685 BCS eligible to participate in the study. Of this pool of individuals, 553 BCS were excluded because they declined or were not interested in participating, or were unreachable by phone. For the remaining eligible participants (*n* = 132), telephone screens were completed to determine study eligibility. For eligible participants, an appointment was made with a project sports medicine physician to obtain medical clearance for participation in the program. Of those who were eligible, 87 attended the medical evaluation. After receiving the medical evaluation, 24 BCS were excluded because the project physician did not approve of their participation in the study or because the individuals subsequently decided not to participate for personal reasons (including not being able to commit to the study or not being interested). After consent was obtained, the 64 eligible and consenting participants were allocated to the two study arms, achieving a sample size of 33 participants in the control group and 31 participants in the intervention group.

Attrition after study enrolment was 15.6% overall (10/64), with six and four participants not completing follow-up in the intervention and control groups, respectively, owing to medical treatments (e.g., eye surgery) (*n* = 3), relocations (*n* = 2), or beginning a new job (*n* = 5). All participants with complete data from the pre- and post-intervention measurements were included in the analyses; thus, the final analytic sample included 25 participants in the intervention group and 29 participants in the control group. Regarding physical activity class attendance rates, 84% of the intervention group attended ≥ 60% of the sessions (15 or more of the 24 sessions). Twenty-five percent of women had family members and friends join at least one session.

### Sociodemographic characteristics

Study participants were primarily educated women (most of them [62.5%] had completed high school or higher education programs), with a mean age of 56.4 years (SD ± 9.5). Half of them were single (50%), and participant households were more likely to be in the middle socioeconomic stratum (62.5%) as opposed to higher (0%) or lower (37.5%) socioeconomic strata. Most of the women (60.9%) were not formally employed, although a large percentage of them worked as caregivers or housekeepers (60.0%). Most of the women received healthcare services through self-paid insurance plans (contributive regimens) (78.1%). When examining the participant’s history of cancer, the average time since cancer diagnosis was 6.4 years (± 5.6). Women from the intervention and control groups did not differ significantly on any of the variables measured at baseline (Table 1).

**Table 1:** Baseline individual characteristics by group from the “My body, My Rhythm, My Voice” study

Characteristic	Intervention (***n*** = 31)		Control (***n*** = 33)	
	Mean/n	SD/%	Mean/*n*	SD/%
Age (years)	57.02	8.70	55.84	10.32
*Marital status*				
Single, divorced, or widowed	15	48.39	17	51.52
Living with a partner or married	16	51.61	16	48.48
*Socioeconomic level*				
Low	15	48.39	9	27.27
Middle	16	51.61	24	72.73
*Education*^a^				
Less than high school	13	41.94	11	33.33
High school graduate	4	12.90	5	15.15
Higher education	14	45.16	17	51.52
*Employment status*^b^				
Not working for payment	18	58.06	21	63.64
Working for payment	13	41.94	12	36.36
*Type of social security*				
Contributive (employer-based)	23	74.19	27	81.82
Subsidized	8	25.81	6	18.18
Time since diagnosis (years)	5.88	4.09	6.97	6.78

Values are presented as mean and standard deviation, or number and percentage

*p value for significance of baseline differences between groups, as tested using *χ*^2^ (categorical data) or *t* test (continuous data)

^a^Education: less than high school to some/no high school. Higher education—technical/vocational program, college degree, graduate degree

^b^Employment status: not working for payment—retired, unemployed, homemaker. Working for payment—employed full-time, employed part-time, freelancer

### *My Body* intervention effects

#### Changes in physical activity levels using accelerometry

From baseline to 8-week post-test, significant improvements were noted in average minutes of accelerometry-derived moderate to vigorous physical activity (MVPA) per day for the intervention versus the control group (+8.99 vs −3.71 min) (Table 2). Based on the subsample of women randomly chosen to wear accelerometers during physical activity sessions, women in the intervention group were recorded as having an average of 13.47 MVPA minutes per session. Online resource 2 shows MVPA records for the physical activity intervention sessions.

**Table 2:** Evaluated outcomes by group from the study My body, My Rhythm, My Voice

Variables	Intervention group				Change Score	Control group				Change Score	Adjust effect^b^		
	Baseline^a^		Post-intervention^a^			Baseline^a^		Post-intervention^a^					
	Mean	SD	Mean	SD	Post.-Bas.	Mean	SD	Mean	SD	Post.-Bas.	Coefficient	Confidence Interval 95%	
**Physical activity (accelerometry)**													
Average sedentary time per week (hours per day)	12.53	4.01	12.51	4.50	0.26	10.58	3.90	10.39	3.77	−0.19	0.05	−1.02	1.13
Average time of MVPA per week (minutes per day)	24.19	16.25	33.18	21.17	0.24	33.46	19.97	29.74	17.73	−3.71	13.01	3.33	22.69
**Self-regulation in physical activity (BREQ-3)**					0.31								
Intrinsic regulation	3.39	0.98	3.84	0.22	0.21	3.79	0.44	3.74	0.36	−0.05	0.50	0.11	0.89
Integrated regulation	3.38	0.94	3.64	0.51	−0.39	3.69	0.45	3.62	0.60	−0.07	0.33	−0.06	0.71
Identified regulation	3.65	0.64	3.89	0.27	0.26	3.85	0.29	3.82	0.37	−0.03	0.27	0.02	0.53
Introjected regulation	1.06	0.97	1.37	0.93	0.24	0.96	0.79	1.16	1.05	0.21	0.10	−0.42	0.63
External regulation	0.46	0.62	0.67	0.96	0.31	0.30	0.46	0.60	0.68	0.30	−0.09	−0.46	0.27
Amotivation	0.86	0.96	0.47	0.52	0.21	0.43	0.60	0.37	0.54	−0.06	−0.33	−0.72	0.06
**Quality of life (EORTC QLQ-C30)**													
Global quality of life functioning	82.00	16.26	81.67	16.32	−0.33	81.61	19.21	84.77	17.26	3.16	−3.49	−10.97	3.98
Physical functioning	86.40	10.27	89.07	10.34	2.67	94.71	6.01	92.18	7.98	−2.53	5.20	1.45	8.94
Role functioning	92.00	14.53	91.33	17.43	−0.67	94.83	10.06	95.98	11.49	1.5	−1.82	−9.76	6.12
Emotional functioning	76.33	19.94	77.67	22.27	1.33	84.20	12.27	81.32	16.91	−2.87	4.21	−3.07	11.48
Cognitive functioning	83,33	20,97	85.33	14.69	2.00	89.66	11.28	87.93	14.01	−1.72	3.72	−4.52	11.97
Social functioning	89.33	21.98	88.67	22.42	−0.67	92.53	14.49	92.53	12.27	0.00	−0.67	−13.40	12.07
Fatigue	21.78	16.19	18.67	17.49	−3.11	13.03	16.55	15.33	14.97	2.30	−5.41	−13.14	2.32
Nausea and vomiting	3.33	8.33	4.67	9.03	1.33	1.15	6.19	1.15	4.30	0.00	1.33	−3.65	6.32
Pain	22.67	19.77	22.67	19.77	0.00	12.64	23.42	12.64	23.42	0.00	0.00	Non estimable	
Dyspnea	4.00	11.06	4.00	11.06	0.00	1.15	6.19	1.15	6.19	0.00	0.00	Non estimable	
Insomnia	24.00	26.39	12.00	18.95	−12.00	17.24	21.12	20.69	24.26	3.45	−15.45	−27.00	−3.89
Appetite loss	6.67	13.61	13.33	19.25	667	1.15	6.19	2.30	8.60	1.15	5.52	−2.04	13.07
Constipation	6.67	19.25	8.00	14.53	1.33	10.34	18.05	5.75	12.81	-4.60	5.93	−3.20	15.06
Diarrhea	8.00	17.43	1.33	6.67	−6.67	4.60	14.70	8.05	19.22	3.45	−10.11	−20.65	0.42
Financial difficulties	22.67	31.51	13.33	21.52	−9.33	13.79	24.43	8.05	19.22	−5.75	−3.59	−15.83	8.66
Arm	21.33	20.01	19.11	20.16	−2.22	13.03	17.33	12.64	16.99	−0.38	−1.84	−12.72	9.05
Breast	14.67	18.98	16.67	21.38	2.00	17.82	19.76	12.93	12.32	−4.89	6.89	−3.97	17.74

Values are presented as mean and standard deviation, or number and percentage

*MVPA* moderate to vigorous physical activity

^a^Data based on study participants completing both baseline and the 8-week follow-up

^b^Based on a GEE analysis: *Y*_*ij*_ = *β*_0_ + *β*_1_ × *group* + *β*_2_ × *time* + *β*_3_ × (*group* × *time*)

#### Motivation to engage in physical activity

Significant between-group increases were noted for intrinsic motivation for exercise (0.45 vs −0.05) and for identified regulation (i.e., high self-determination, the behavior is executed because it is enjoyable and of interest to the individual) (0.27 vs −0.03) for the intervention versus the control group (Table 2).

#### Quality of life

The results of the EORTC QLQ-C30 did not show a change in the global score (Table 2). However, there are some specific positive results comparing changes between groups. For the physical functioning subscale scores (+2.67 vs −2.53) and insomnia (−12.00 vs 3.45), the change between the intervention versus control group was statistically significant. Additionally, although statistically non-significant, the intervention group also showed an important difference in gastrointestinal function. Compared with the control group, the intervention group had fewer diarrhea (−6.67 vs 3.45) and constipation (1.33 vs −4.60) complaints. These results indicate a pattern in some symptoms towards higher QoL among the intervention versus the control group. Adjust effect for pain and dyspnea could not be estimated due to changes were not found.

### *My Body* program acceptability

#### Barriers and facilitators to engage in physical activity program

Table 3 provides details of the reported barriers (i.e., time management, limited self-efficacy, limited social support) and facilitators (i.e., enjoyment, resilience skills, peer care network) to engage in the *My Body* program. Of note, in addition to identifying barriers, BCS mentioned having used the following motivational coping strategies: (1) focusing on positive attitudes towards self-care that allow them to satisfy their own needs for enjoyment and personal time and being aware of the benefits of prioritizing their own well-being; (2) using available personal, family, and social resources such as better time management, making agreements with family members to distribute tasks, and speaking to employers to make their working hours flexible so that they could regularly participate in the program; and (3) developing home-based alternatives by recording sessions and sharing videos to practice at home.

**Table 3:** Reported barriers, facilitators, and benefits of *My Body* physical activity program

	Intrapersonal	Interpersonal	Community	Exemplar quotes
**Barriers to engage in*My Body*physical activity program**	Time management: restricted according to medical appointments	Limited social support (family, employers)	Socioeconomic status given the personal competing priorities to invest money and time	*“I say, what more to do than clean the house, sweep, do laundry, go get the kids, the grandkids from school, come back…what more exercise than what I already do.”*
	Limited self-efficacy: not feeling able to dance	Intrahousehold gender disparities to distribute care and home tasks	Lack of physical activity programs offered within the health care system	
		Fears and risk perceptions from family and some healthcare professionals		
**Facilitators to engage in*My Body*physical activity program**	Personal enjoyment through dancing	Group-based physical activity practice enhancing social support represented in program companions (friends, family)	Motivated knowledgeable staff trained to address breast cancer related issues and physical activity	*“I’d think: the cancer, 20 rounds of chemo, 10 rounds of radiotherapies, how would I be able to even lift a finger? Then when I saw myself in this program I thought ‘no way, this is great’. Knowing that not everything is bad, that after all what cancer put me through, I was able to dance again”*
	Positive beliefs regarding physical activity	Guided physical activity changing risk perceptions and strengthening motivation	Interdisciplinary team (sports physicians, psychologists, respiratory therapists) providing safety, respect, and affection	
	The resilience process associated to the disease	Peer care network (emotional support and role modelling)	Location of the sessions was accessible by public transportation	
**Perceived benefits from engaging in*My Body*physical activity program**	Increased energy (vitality) and endurance to perform daily tasks			*“This program truly helps and should be growing even more. I don’t know where to go, if I should go to where I got treatment, to the chemo room and tell them ‘look, there’s a program for this, to help us with our self-esteem because it goes so low’. I’d love to spread the word because I suffered a lot and I don’t want others to go through what I went through. On the contrary, I want to walk into those chemo rooms where people are pitying themselves and tell them, ‘no, we’re going to get through this and get healthy and pretty’.”*
	Decreased pain and recovery of movement for the arms, knees, hips, and feet			
	Improvements in flexibility, joint mobility, coordination, and agility			
	Improvements in the sleep cycle			

#### Perceived benefits of the program

According to the BCS, *My Body* enabled positive changes in their perceptions of physical activity. A number acknowledged that, instead of causing discomfort, exercise creates wellness. Some of the women described how they had believed that daily tasks and housekeeping chores provided a sufficient physical activity to maintain optimal health. They reported that through engaging in the *My Body* program they viewed physical activity during leisure time as a pleasant habit of personal enjoyment, which can be better maintained using the self-regulation skills that they were taught as part of the physical activity program.

Benefits primarily reported by BCS as part of the semi-structured interviews were related to a better perception and management of their body and rising awareness and control over their movements and capabilities (Table 3). Some participants who said that they tended to feel isolated, highlighted improvements in their social skills and connectedness. They described a shift from relating to their bodies from a place of fear, to feeling capable and comfortable in their bodies. The program enabled positive attitudes (e.g., discipline, personal agency, self-confidence) and improved self-image and mood, and motivation to leave home. In general, the narratives of the participants indicated that the physical activity dance sessions enabled changes in their self-belief systems, which contributed to the strengthening of the following outcomes: (1) self-care, by acquiring a space for personal enjoyment; (2) self-efficacy, by feeling capable of generating changes in important health behaviors; (3) self-esteem, by generating a sense of worth and pride, and celebrating their own achievements; and (4) self-concept, by recognizing their own physical, psychological, and social capacities, and valuing positive behaviors such as personal coping strategies and empathy towards others.

#### Program recommendations

During the post-program interviews, the intervention women underscored the following areas as successful aspects of the program: (1) the professionals implementing it (instructors, members of the research team), as they encouraged participants within a context of worth and trust; (2) the sessions, which were perceived as innovative, attractive, challenging, and fun; (3) practicing physical activity with peers, as they could identify with each other’s experiences and provide/receive support; (4) being able to invite family members and friends to join the program sessions and share a fun space; and (5) the communication channels created between the research team and the participants, such as WhatsApp, which generated a feeling of worth and allowed access to recorded videos of the sessions. Regarding suggested program improvements, a few participants mentioned the location was too far from their home, some said the time schedule was inconvenient for people with working schedules, and some expressed the nutrition workshops should include practical sessions such as recipe preparation. Also, some BCS expressed willingness to perform as physical activity promotors whether disseminating the program with other BCS or facilitating its implementation in other cities, using their word of mouth to communicate experienced benefits. Table 4 summarizes issues and lessons learned about the intervention by triangulating quantitative and qualitative results.

**Table 4:** Integration of quantitative and qualitative results from the study My body, My Rhythm, My Voice

Program outcomes		Quantitative measures		Qualitative measures		Lessons learned
Engagement of breast cancer survivors	Recruitment	Recruitment rate	64/553	Thematic analysis of reported barriers to engage in physical activity	“A lot of the time you stop yourself from doing something because of the fears that others instill in you. (…) So many people do this, they tell you ‘oh you can’t do this with your pain, you can’t warm up’ no, no, no, you can’t do anything no, no, no. (…) I started researching and it turns out that when you get surgery you have to start exercising right away unlike what other people tell you. It’s important for Doctors to become aware of this, not just tell you no, no, no, they should motivate you instead”. (Intervention group, interview #3).	Recruitment of a diverse group of women was difficult due to misperceptions of physical activity and its potential benefits during and post cancer. For future interventions, it is relevant to engage health care professionals and providers as physical activity promotion agents.
	Participant characteristics	Middle socioeconomic level	62.17%	Thematic analysis of BCS narratives	“I’d say: it doesn’t matter, if I have to make sacrifices, I’m going to do it, I’m going to finish [the intervention] and that’s what I did and now I feel fulfilled to have been able to do it, even if I had to sacrifice a lot, because I’m always thinking about everyone else and not myself.” (Intervention group, interview #5)	Relevant background characteristics of the BCS who participated in *My Body* include their socioeconomic level, employment status, medical history, and time management practices regarding personal competing priorities (i.e., self-care, household caregiving duties, work, medical schedule).
		Not working for payment	60.85%			
		Overweight/obese	80%			
		Time since diagnosis (years)	5.43			
	Participant retention	Attendance to ≥60% of *My Body* sessions	84.40%	Thematic analysis of facilitators to engage in physical activity.	“I felt fulfilled with everything we did and all the dancing (...) it was my exit from home. I used to spend all day lying down. This has been very exciting and beautiful for me. I tell everyone in my house that I'm sorry because it's over.” (Intervention group, interview #8). “It motivates you to know that it’s led by people who know what they’re doing. They’re always looking out for you, asking if you got back safe or why you’re not there yet. The attention that they give us by asking how we’re doing, how things are going, how did we do, that’s something you sometimes don’t even get from family.” (Intervention group, interview #13).	The high retention rate among the Intervention group after starting *My Body* program might be related to facilitators reported at all levels: individual (enjoyment through dancing, positive beliefs regarding physical activity, the resilience process associated to the disease), interpersonal (group-based guided physical activity practice, peer care network), and community (motivated knowledgeable staff, interdisciplinary team, accessible place).
Effects of the intervention among breast cancer survivors in the physical activity group	Change in mean physical activity levels	Average time of MVPA per day	8.99 min	Thematic analysis of BCS perceived benefits	“I felt really good because honestly, my leg would give up on me a lot before. I was going to get surgery but with this I’ve felt much better, I can walk now, and I feel like I’ve been reborn. I’ve walked a lot and already feel so good.” (Intervention group, interview #5) “Learning to know our bodies has been important too. Learning to know our own body and its rhythm. If I couldn’t do something, then it was ok and when I could do it, it was at my own pace.” (Intervention group, interview #1)	As a result of the intervention, BCS on average added 9 minutes to their average time of MVPA per day, when compared with the control group.
	Change in motivation to engage in physical activity	Intrinsic regulation score	0.45	Thematic analysis of BCS’ perceptions of physical activity	“I learned that you’ve got to let go of the idea that you can’t do something. I used to think I couldn’t dance, and it turns out I can, and it makes me happy. You really need to free yourself from all those things that are holding you back.” (Intervention group, interview #6) “Learning to have discipline and will power. For example, there were 3 classes left and I had all this leg pain, but I’d say no, I have to do this and finish it." (Intervention group, interview #4) “I was a dancer as well (…). So, I’d think: the cancer, 20 rounds of chemo, 10 rounds of radiotherapies, how would I be able to even lift a finger? Then when I saw myself in this program I thought ‘no way, this is great’. Knowing that not everything is bad, that after all that cancer put me through, I was able to dance again. I love dancing” (Intervention group, interview #2)	The increases in the intrinsic motivation to engage in physical activity among BCS were possibly related to the experienced changes towards perceiving physical activity as a pleasant habit of personal enjoyment and self-care, positive attitudes (e.g. discipline, will, self-confidence), improving self-image and mood, and motivation to leave home among their perceptions of physical activity as an enjoyable behavior and perceived broad benefits (e.g., impacts in the self-belief system, improvements in flexibility, joint mobility, coordination, and vitality).
	Change in quality of life	Quality of life score		Thematic analysis of BCS perceived benefits	“In an emotional level I really liked it because it helped me stay in a more optimistic state of mind (...) here I feel identified with everyone. I worked hard, I was very happy, and I felt that I forgot all my problems” (Intervention group, interview #3).	We did not find a significant change in QoL, but through the interviews women reported broad perceived benefits contributing to well-being enhancement. It is necessary to evaluate a larger sample size and longer intervention to capture changes in QoL. Additionally, relevant health outcomes for BCS that should be reported include sleep dysfunction, joint pain, specific self-efficacy measures for dancing (or corresponding type of physical activity), coordination, time management, self-care, and social networking.
Considerations for future implementation	Perceived acceptability of *My Body* program			Thematic analysis of aspects to maintain	“For me it was very gratifying [sharing with BCS] not because it happened to them but because I didn’t feel so alone. It’s not the same to talk to or be with someone who hasn’t been through what I’ve been through than to have a fellow fighting companion that knows what it’s like.” (Intervention group, interview #6)	Women underscored as successful aspects of the physical activity program: (1) the professionals implementing it, (2) the enjoyable physical activity sessions, (3) practicing physical activity with peers, family members, and friends, and (4) the communication channels.
				Thematic analysis of aspects to strengthen or include	“These programs truly help and should be growing even more because if you tell us where to go, that’s where we’ll go to promote it further (…) tell them ‘look, there’s a program for this, to help us with our self-esteem because it goes so low’. I’d love to spread the word because I don’t want others to go through what I went through. On the contrary, I want to walk into those chemo rooms were people are pitying themselves and tell them, ‘no, we’re going to get through this and get pretty’.” (Intervention group, interview #10)	BCS suggested (1) increasing sites and hours for the physical activity sessions, (2) including nutrition workshops for preparation of recipes, and (3) installing capacity among BCS to become agents of physical activity promotion.

## Discussion

*My Body*, a theory-driven and dance-based physical activity program for BCS, has the potential of being effective for increasing physical activity and improving quality of life by using a community setting approach. The intervention group was able to add ~10 min of MVPA per day (i.e., ~70 min/week) in response to the intervention. Physical activity has been linked with a variety of positive health outcomes in adults, including women who are survivors of breast cancer [10, 46]. Importantly, we observed an increase in intrinsic regulation for physical activity, reflecting the fact that women became increasingly motivated to participate in regular physical activity based on the benefits that it could bring to them personally. Regarding QoL, we observed improvements in the scores for physical functioning and insomnia symptoms—two key outcomes for BCS. Additionally, through the post-intervention interviews, women reported broad perceived benefits contributing to well-being enhancement. The cancer-specific focus of the program, their enjoyment of the reasonably moderate physical activity intensity, and the encouraging program environment are positive features that could be maintained in the *My Body* program. Lastly, adherence to the program was high. To the best of our knowledge, this is the first study in Latin America to engage a transdisciplinary community-setting approach to implement a safe, accessible, and culturally appropriate program to affect the general wellness of BCS. While the study was conducted prior to the onset of the COVID-19 pandemic, participants also appreciated the availability of recorded sessions, with remote platforms such as Facebook and YouTube serving as additional methods for delivering portions of the program during times when in-person gatherings are limited.

In the last few years, several studies in high-income countries (HIC) have reported the effects of dance-based physical activity interventions on BCS, reporting promising improvements in physical, mental, and social health [47, 48]. Given this, this research area can benefit at this juncture from the delivery and evaluation of effective intervention protocols at a community level [49]. *My Body* was effectively delivered as part of a publicly available municipal physical activity program offered by the city of Bogotá. Future larger-scale studies involving randomization or crossover designs with larger periods of intervention and evaluation could provide evidence of program effects on additional important outcomes for this population. Furthermore, follow-up of a longer duration could address the question of sustainability.

Across different cultures, dancing has been indicated as providing an optimal balance between an effective as well as engaging training protocol for women, including those with a breast cancer diagnosis [48]. This was reflected in the self-regulation for physical activity increases, particularly, significant increases for intrinsic motivation and identified regulation which, according to self-determination theory, are associated with adherence to regular active behaviors [42, 50]. These results suggest that participants were no longer exercising simply for extrinsic reasons but developed a more personal interest in physical activity, finding it enjoyable and beneficial [41]. Women expressed interest in replicating active behaviors by dancing along with videos at home and noting interest in becoming champions for physical activity among BCS more generally. Additionally, BCS underscored the peer care network as a facilitator for engaging in the intervention, which generated feelings of comfort and confidence, and enabled emotional support and role modeling to support one’s own goals and dissipate personal fears. Overall, participants expressed that group-based cancer-specific physical activity encouraged a sense of belonging, worth, mutual support, and emotional bonding [51].

In terms of cancer survivors’ QoL, a growing body of literature has indicated that tumor-related peer support groups enhance a long-term development of positive QoL and coping [51–53]. Future studies should involve larger samples and longer evaluation periods to potentially capture additional changes in other symptoms and dimensions of QoL.

Analyzing participant narratives allowed for a qualitative process evaluation of changes in women’s perceptions about physical activity. Women reported that they gained confidence in their competence to perform physical activity as a possible additional activity to daily tasks and found it to be increasingly relevant to personal enjoyment, self-care, and well-being enhancement. Such improvements in participant attitudes indicate the potential of *My Body* to improve perceived behavioral capabilities to be active.

Overall, participants were satisfied with the program while identifying facilitators and barriers that align with findings obtained in similar studies [52, 53]. Facilitators of program attendance included enjoyment of dance sessions, increased social support, and motivated and knowledgeable staff. The main reported barriers to regular attendance were medical schedules (as patients and caregivers) and limited social support among family. Some participants missed sessions due to medical appointments, underscoring that the lack of physical activity programs offered within the health care system is a community-level barrier. If physical activity promotion was part of their oncological treatment, then the overlap between medical appointments and activity sessions could potentially be more readily diminished.

Future studies should include virtual sessions that could improve adherence. Women stated having improved skills for time management and negotiation among their social networks related to distributing home tasks and scheduling appointments. Indeed, previous studies have indicated that not only women need to be encouraged to see beyond the myths and barriers associated with physical activity in BCS, but family members and even physicians could benefit from these insights, as well [41, 54]. Our findings support this idea, as the underlying fears related to BCS performing physical activity could be seen not only during the intervention but especially before the program even started. Such barriers included several difficulties in accessing information from the health care setting relating to potential participants, and some ethics committee fears concerning approving the intervention due to perceived risks to the participants, which were not justified given the substantial evidence base supporting the benefits of such programs for BCS.

### Strengths and limitations

In relation to study strengths and limitations, our multidisciplinary mixed-methods approach allowed the development of an evidence-supported, culturally relevant, and attractive behavior change intervention (i.e., a dancing protocol). However, participants noted that they also would have appreciated receiving complementary workshops regarding a healthy diet. Second, recruitment was found to be challenging in this first-generation study, particularly given some hesitation among healthcare providers to recommend physical activity to BCS, even though evidence strongly supports its benefits for this population. Nevertheless, partnerships with multisectoral institutions and the sports physician evaluation were crucial for women feeling supported in enrolment. We subsequently reported these results to clinicians and the ministry of health, creating trust and feasibility for future studies with BCS. Additionally, we learned that relevant health and behavioral outcomes for BCS that should be reported include sleep dysfunction, joint pain, specific self-efficacy measures for dancing (or corresponding types of physical activity), time management, self-care, and social networking. Likewise, for future studies involving larger samples, the analysis could benefit from being stratified by time since diagnosis, as this can be an important modifier of the intervention effects [22]. Although we confirmed the relevance of including certain portions of the health sector, the sports/recreation sector, and academia in this research, further dissemination of the program should consider the direct participation of local oncologists to recommend the community-based program.

## Conclusion

*My Body* is a theory-driven community-based physical activity program for BCS implemented through the engagement of multi-sectoral stakeholders. It has the potential of generating behavioral changes while making use of the benefits of dance sessions as a catalyst for engaging in health-enhancing physical activity. It was tailored specifically to a real-world community setting and to meet BCS needs regarding physical, mental, and social health. We believe that the promising results found in this first-generation study in Latin America merit additional investigation in relation to this important and growing population segment in Colombia.

## Supplementary Information

**Additional file 1. **Components of *My Body* behavioral intervention.

**Additional file 2. **Physical activity intensity during *My Body* intervention dancing-based sessions.

## Data Availability

The datasets used and/or analyzed during the current study are available from the corresponding author on reasonable request.

## Abbreviations

BPM: Beats per minute
BMI: Body mass index
BCS: Breast cancer survivors
CI: Confidence intervals
EORTC QLQ-C30: European Organization for Research and Treatment of Cancer Quality of Life Questionnaire
GEEs: Generalized estimating equations
HR: Heart rate
*My Body*: “My body, My Rhythm, My Voice”
QoL: Quality of life
SD: Standard deviation
SDT: Self-determination theory
