# Supplementary material for: “My Body, My Rhythm, My Voice”: a community dance pilot intervention engaging breast cancer survivors in physical activity in a middle-income country
Source: Pilot Feasibility Stud. 2023 Feb 28;9:30. doi: 10.1186/s40814-023-01253-x (PMC9971676; doi:10.1186/s40814-023-01253-x)
Supplement: Supplementary file 1 — Additional file 1. Components of My Body behavioral intervention. [file 40814_2023_1253_MOESM1_ESM.pptx]

## Slide 1
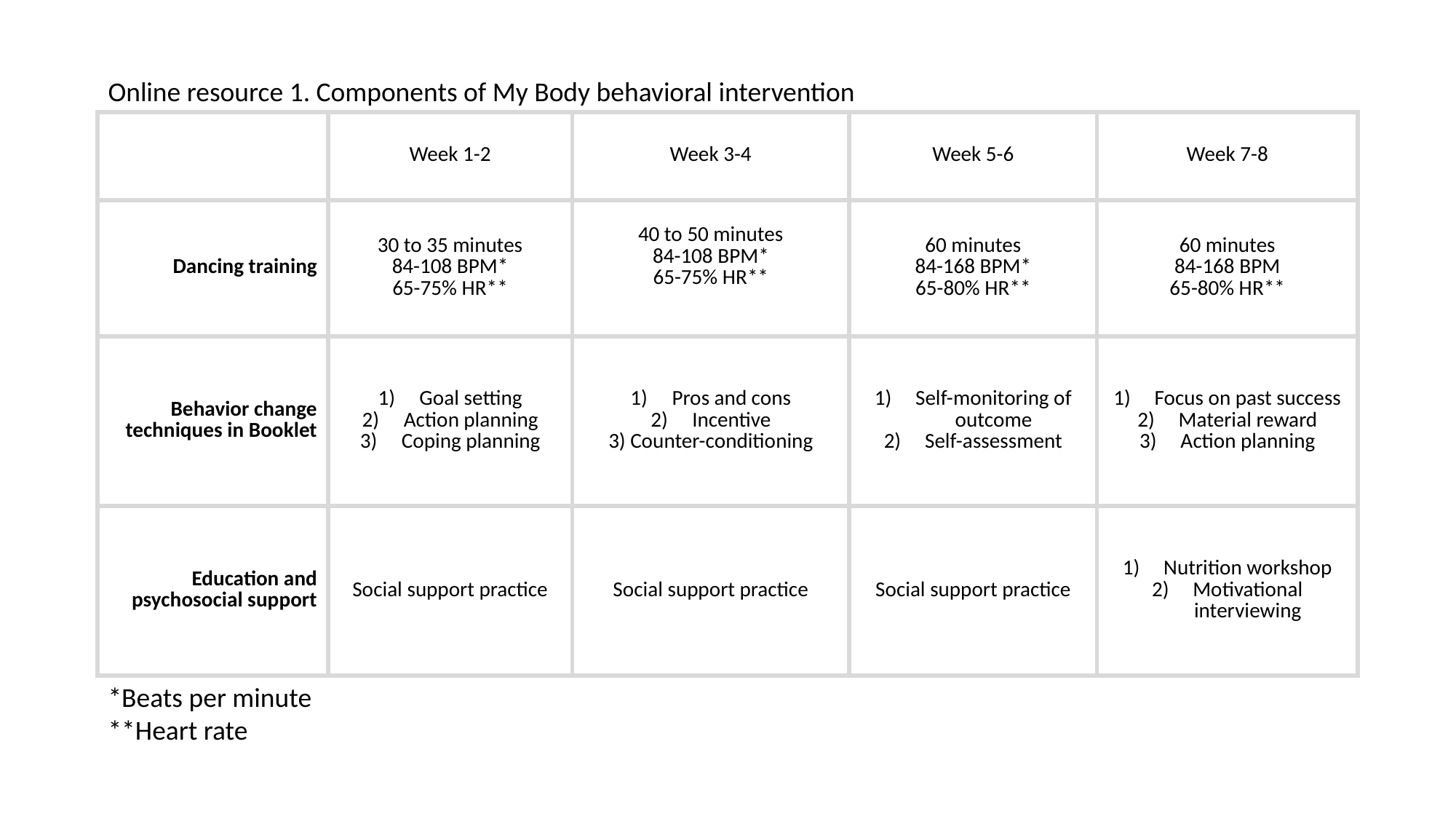

Online resource 1. Components of My Body behavioral intervention
| | Week 1-2 | Week 3-4 | Week 5-6 | Week 7-8 |
| --- | --- | --- | --- | --- |
| Dancing training | 30 to 35 minutes 84-108 BPM\* 65-75% HR\*\* | 40 to 50 minutes 84-108 BPM\* 65-75% HR\*\* | 60 minutes 84-168 BPM\* 65-80% HR\*\* | 60 minutes 84-168 BPM 65-80% HR\*\* |
| Behavior change techniques in Booklet | Goal setting Action planning Coping planning | Pros and cons Incentive 3) Counter-conditioning | Self-monitoring of outcome Self-assessment | Focus on past success Material reward Action planning |
| Education and psychosocial support | Social support practice | Social support practice | Social support practice | Nutrition workshop Motivational interviewing |
*Beats per minute
**Heart rate
