# Supplementary figures and images for: “My Body, My Rhythm, My Voice”: a community dance pilot intervention engaging breast cancer survivors in physical activity in a middle-income country
Source: Pilot Feasibility Stud. 2023 Feb 28;9:30. doi: 10.1186/s40814-023-01253-x (PMC9971676; doi:10.1186/s40814-023-01253-x)

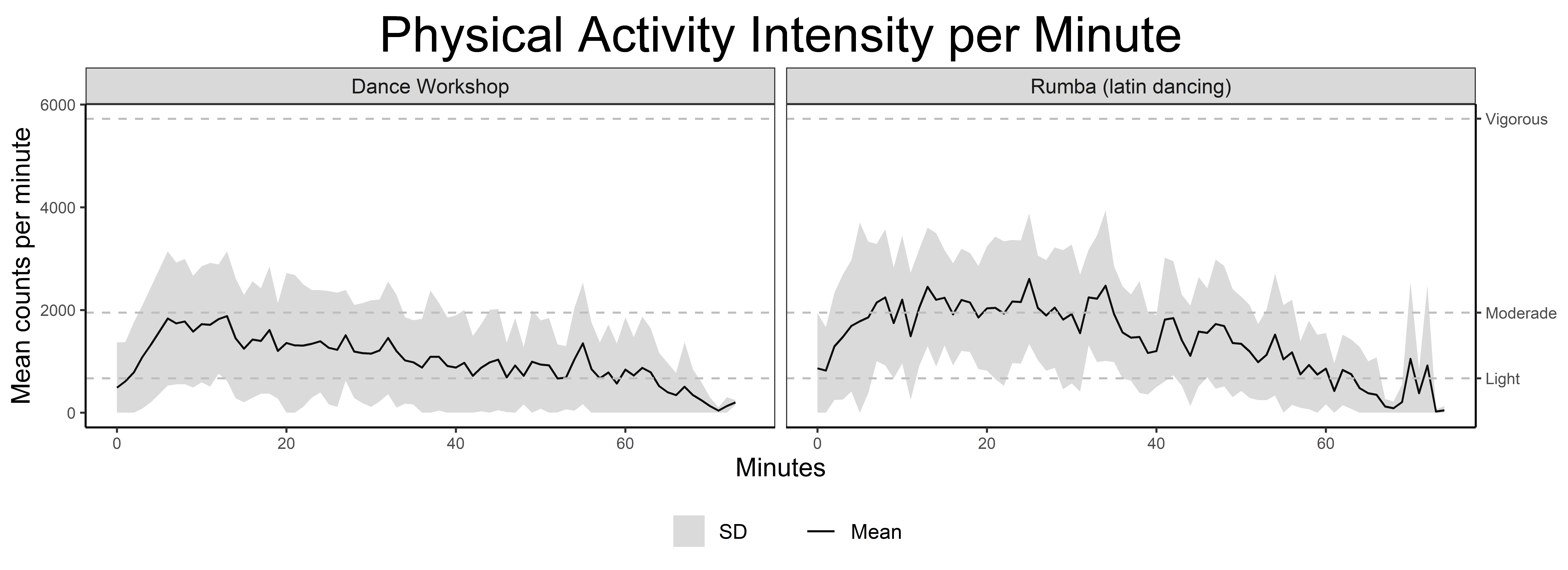

Supplement: Supplementary file 2 — Additional file 2. Physical activity intensity during My Body intervention dancing-based sessions. [file 40814_2023_1253_MOESM2_ESM.tiff]
